# Integrating Phosphorylation Network with Transcriptional Network Reveals Novel Functional Relationships

**DOI:** 10.1371/journal.pone.0033160

**Published:** 2012-03-14

**Authors:** Lin Wang, Lin Hou, Minping Qian, Minghua Deng

**Affiliations:** 1 Center for Theoretical Biology, Peking University, Beijing, China; 2 Key Laboratory of Mathematics and Applied Mathematics, School of Mathematical Sciences, Peking University, Beijing, China; 3 Center for Statistical Science, Peking University, Beijing, China; University of Sheffield, United Kingdom

## Abstract

Phosphorylation and transcriptional regulation events are critical for cells to transmit and respond to signals. In spite of its importance, systems-level strategies that couple these two networks have yet to be presented. Here we introduce a novel approach that integrates the physical and functional aspects of phosphorylation network together with the transcription network in *S.cerevisiae*, and demonstrate that different network motifs are involved in these networks, which should be considered in interpreting and integrating large scale datasets. Based on this understanding, we introduce a HeRS score (hetero-regulatory similarity score) to systematically characterize the functional relevance of kinase/phosphatase involvement with transcription factor, and present an algorithm that predicts hetero-regulatory modules. When extended to signaling network, this approach confirmed the structure and cross talk of MAPK pathways, inferred a novel functional transcription factor Sok2 in high osmolarity glycerol pathway, and explained the mechanism of reduced mating efficiency upon Fus3 deletion. This strategy is applicable to other organisms as large-scale datasets become available, providing a means to identify the functional relationships between kinases/phosphatases and transcription factors.

## Introduction

Living cells sense and respond to the changing environment through efficient signaling pathways, driven by phosphorylation events acting in concert with transcriptional regulation to transmit and process the signals. In this process, the major players are kinase, phosphatase, and transcription factors (TFs). A comprehensive understanding of the organizing principle of the signaling network, including the molecular function of each protein, the cooperation between different molecules, and the mechanisms by which the pathways are selected and regulated, requires a multi-tier description of the underlying networks.

The interactions involved in the signaling network are protein-protein interactions between kinase/phosphatase and substrates, and protein DNA interactions between TF and target genes. Simply stated, the signaling network is a combination of two fundamental networks - a phosphorylation network and a transcriptional regulatory network. In the model organism *S.cerevisiae*, the above interactions have been experimentally characterized in a high-throughput fashion from both biochemical and genetic perspectives. Kinase-substrate interactions were detected by *in vitro* proteome chip technology, and individual phosphorylation events were assembled into a phosphorylation map for *S.cerevisiae* [1]. The protein-DNA interactions were measured by ChIP-chip experiments (chromatin immunoprecipitation coupled with DNA chip) [2] [3]. These biochemical and physical interaction maps form a static scaffold of the signaling network through which signals flow. However, these datasets provide limited insight with regards to the functional links within and between pathways.

Complementary to the physical interaction datasets, genetic approaches which study the mRNA expression levels when cells are perturbed provide a functional view of the cellular system. In the budding yeast, using whole genome mRNA expression as a phenotype, the phenotypic change upon single kinase, phosphatase [4], or TF [5] deletion was measured, which revealed the transcriptional changes in response to perturbations to the signaling network. The set of up and down regulated genes forms what is called a signature corresponding to the perturbed protein. A set of proteins together with their signatures constitute a functional network. We expect that integrating physical interaction networks with functional networks will derive a better picture of the structure and function of the signaling network.

In spite of the close relationship between phosphorylation and transcriptional regulation, the two networks are generally investigated separately. Many experimental and computational approaches endeavor to disclose the pair-wise interactions of both networks [1] [2] [6] [7] [8] [9] [10]. However, integrative studies of the two networks are very limited. A recent study generated a first-generation phosphorylation map for *S.cerevisiae*, and integrated the phosphorylation results with transcription factor binding data [1]. The results demonstrated that the largest class of kinase substrate is transcription factors, and revealed several new regulatory modules. Another study demonstrated in the cell cycle process, cyclin-dependent protein kinase (CDK) and transcription factors commonly form feed forward loops to activate different phases of the cell cycle [11]. In these studies, kinase/phosphatase and transcription factor are connected via biochemical interactions. Although such approaches have shed light on the prevalent regulatory motifs in the signaling network, the functional link between these regulators cannot be inferred. In this study, we propose to link kinase/phosphatase and transcription factor by using the transcriptome as an anchor.

In order to integrate the heterogeneous networks, a prerequisite is to understand the property of individual networks and to address the commonalities and distinctions between them. To explain heterogeneous networks in a unified framework, we performed a systems approach by studying network motifs in different networks. The results show that the phosphorylation and transcriptional regulatory networks employ different network motifs to achieve their unique functions. This finding supports the idea that network motifs are building blocks of cellular systems. Inspired by the above analysis, we defined a hetero-regulatory similarity score to integrate the phosphorylation network and transcriptional regulatory network and to identify hetero-regulatory modules through the integrative approach. The predicted modules successfully recovered the MAPK pathways in *S.cerevisiae*, and also shed light on the cross-talks between different MAPK pathways. The utility of this integrative approach is also confirmed through two novel findings that come out of an in-depth examination of the heterogenous regulatory modules. This includes predicting novel function of transcription factor Sok2 and presenting an explanation for the reduced mating efficiency that results upon deletion of Fus3.

## Results

### Co-functional prediction suggests distinct regulatory pattern between the phosphorylation and transcriptional networks

**Figure 1:**
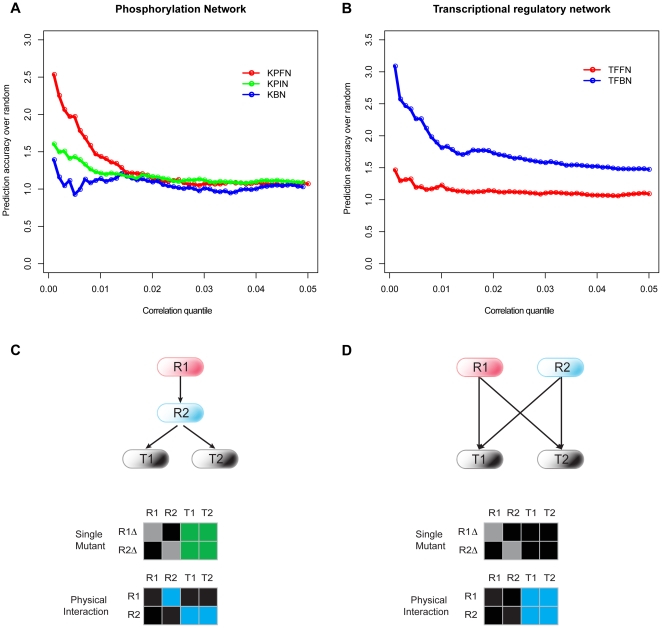
Co-function prediction using different datasets suggests distinct regulatory pattern in phosphorylation network and transcriptional network. Shown is the fold change of prediction accuracy using different datasets compared with random levels (the fraction of co-function gene pairs in relevant network). (A) Comparison in phosphorylation networks, KPFN (functional network derived from a microarray study of kinase/phosphatase single deletion strains), KBN (biochemical network derived from in vitro protein chip), and KPIN (physical network of kinase/phosphatase interaction). (B) Comparison in transcriptional regulatory networks, TFBN (transcription factor binding network derived from ChIP-chip experiments) and TFFN (functional networks derived from transcription factor single deletion strains). (C) A linear regulatory model. Regulators R1 and R2 function in a linear regulatory pathway, and T1 and T2 are their targets. R1 and R2 share similar profiles in functional network, but disparate profiles in physical network. (D) A parallel regulatory model. Regulators R1 and R2 function in a parallel regulatory pathway, and T1 and T2 are their targets. R1 and R2 share similar profiles in physical network. However, they have no interaction in functional networks due to genetic buffering. Grey: unobserved data; Green: functional interaction; Blue: physical interaction; Black: no interaction.

Since various networks of phosphorylation and transcriptional regulation are available, a straightforward question is to test how well different datasets can recapitulate current biological knowledge. We used five datasets to predict co-functional gene pairs, and assessed the accuracy by comparing the predictions with a gold standard dataset (see Materials and methods). The datasets covered both genetic and biochemical aspects of phosphorylation network and transcriptional regulatory networks, including KPFN (functional networks derived from a microarray study of kinase/phosphatase single deletion strains [4]), TFFN (functional networks derived from TF single deletion strains [5]), KBN (biochemical networks derived from *in vitro* protein chip [1]), KPIN (physical networks of kinase/phosphatase interaction [10]), and TFBN (TF binding network derived from ChIP-chip experiments [12] [13]). Except for KPIN, the other networks are directed. In each network, the similarity between regulators is calculated by the Pearson correlation coefficient of their interaction profiles, which measures the extent two regulators share common targets. It is expected that highly correlated pairs are co-functional, however the prediction accuracy varies a lot across the five networks considered (Figure 1 A, B). In phosphorylation network, functional networks (KPFN) are more predictive than biochemical or physical interaction networks (KPIN, KBN); while in the transcriptional regulatory network the opposite is true.

Apart from our observation, in transcriptional regulatory network, it has been pointed out in literature that TF signatures overlap poorly with their corresponding binding targets [5], possible explanations of which include protein-protein interactions between TFs [14], homology relationships [14], and indirect transcriptional regulation [5]. Our data and other studies indicate a substantial discrepancy between the biochemical networks and functional networks; explaining this contradictory behavior is an interesting question that we will address below.

We present a simple model to explain the difference in genetic signature and biochemical interaction profile. If two regulators act in a linear pathway (Figure 1C), the deletion of either one will cause the same effect, thus lead to similar signatures. However, their binding targets may vary. In contrast, if two regulators work in parallel (Figure 1D) and they bind to the same targets, the deletion of either one will have no effect on the expression level of target genes due to genetic buffering. As a result, they have similar biochemical interaction profiles but distinct signatures. Hence, we hypothesize that the regulatory motifs in the phosphorylation and transcriptional regulatory networks are different, with phosphorylation networks being abundant with linear pathways and transcriptional regulatory network abundant with parallel pathways.

### Phosphorylation network and transcriptional network differ in motif usage

**Figure 2:**
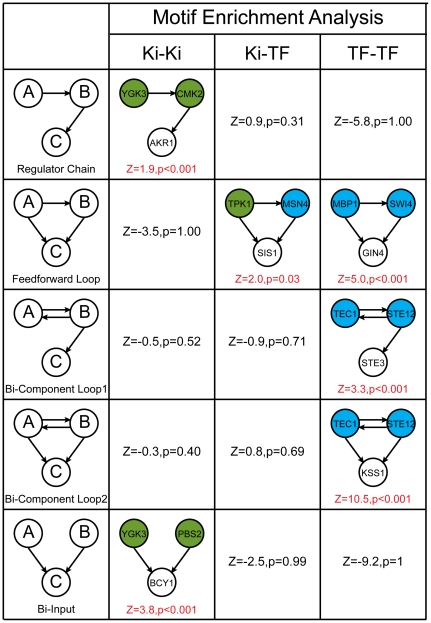
Motif enrichment analysis reveals different motif usage in the phosphorylation and transcriptional regulatory networks. Five regulatory motifs were investigated in three networks, phosphorylation network, transcriptional regulatory network, and the combined network. Node A and B represent the regulators (kinase/phosphatase or transcription factor), and node C represents the target gene. In the combined network, node A represents kinase/phophatase and node B represents a transcription factor. P-values and Z-scores are calculated based on a randomly shuffling process (see Materials and methods). For the enriched motifs, an example from the corresponding network is provided.

To validate our hypothesis that phosphorylation network and transcriptional regulatory network are abundant with different motifs, we examined the network motifs of KBN, TFBN, and their combination. We excluded KPIN because it lacks the direction between two kinase. In these networks, nodes represent regulators and targets, and the edges are directed, representing the physical binding of a regulator to certain targets. In order to investigate the cooperative pattern between regulators, we enumerated three node motifs with the restriction that the two regulators have a direct or indirect regulation on the target gene. By calculating the occurrence of the motifs and contrasting with randomly shuffled networks (see Materials and methods), the significance of network motifs is evaluated (Figure 2).

Our data demonstrates phosphorylation network and transcriptional regulatory network utilize different network motifs. The motif “regulator chain” is only enriched in phosphorylation network. Based on the linear regulatory model (Figure 1C), it can be inferred that KPFN is more predictive of co-function than KBN, which coincides with our observation. The “bi-input” motif is also enriched in phosphorylation network, resulting in a genetic buffering effect of phosphorylation events [4]. In transcriptional regulatory networks “feed forward loop” (FFL) motif was enriched, which have already been widely discussed [2] [15] [16]. The motifs with loop structure within regulators (bi-component loop1, bi-component loop2) are also enriched in transcriptional regulatory networks. In these motifs, two TFs transcribe each other, and generate a bi-stable system, which switches between two alternative states [17] [2]. The two motifs tend to characterize an important mode in transcriptional regulation. TFs cooperate to regulate a set of genes (bi-component loop 2), but their functions are not completely redundant (bi-component loop 1). For example, Ste12 and Tec1 are two TFs that co-regulate genes in filamentous pathway (for example, Kss1), but only Ste12 activates genes in mating pheromone pathway (for example, Ste3) (Figure 2). In this case, the resultant signatures are divergent but their binding profiles overlap with each other on the co-regulated genes (Figure S1). This phenomenon is termed mixed epistasis in phosphorylation network [4], where two kinase partly buffer each other, and also have unique functions themselves. We demonstrate that this definition can also be extended to transcriptional network according to the enriched bi-component loops. Because of the enriched buffering relationships in transcriptional regulatory networks, TFBN is more predictive of co-function than TFFN, which is also consistent with our observation. In the combined network, the enriched motif is FFL, which couples phosphorylation with transcription. A previous study showed that FFL formed by kinase CDK1 and transcriptional factors was important to drive temporal transcriptional responses in cell cycle regulation [11].

It is noted that the motifs enriched in the phosphorylation networks are completely disjoint from those in the transcriptional regulatory networks, which suggests the two networks are structurally quite different. Our results also support the idea that motifs are indeed building blocks of biological networks, and different usage of network motifs carries out distinct function. Except for motif usage, the difference might also be due to different global topological features of KBN and TFBN. However, this possibility is not strongly supported as in both KBN and TFBN the degree distributions obey a power law form (Figure S2), and their edge densities are at comparative levels (0.035 in KBN vs. 0.030 in TBN).

### Hetero-regulatory modules couple phosphorylation with transcriptional regulation

**Figure 3:**
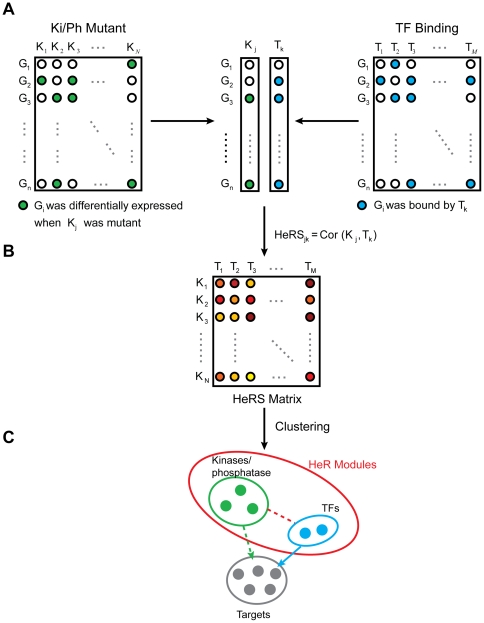
Strategy of identifying hetero-regulatory (HeR) modules. (A) Calculation of hetero-regulatory similarity (HeRS) score, which is the Pearson correlation coefficient of the functional profile of a kinase and the physical binding profile of a transcription factor. (B) Hierarchical clustering of hetero-regulatory similarity (HeRS) matrix. (C) A toy example of hetero-regulatory module, which is composed of a group of kinases/phosphatases, a group of transcription factors, and their target genes.

Comprehensive techniques that individually analyze phosphorylation network and transcriptional regulatory network have been extensively studied [1] [11], however, an integrative method is still lacking in literature. To our knowledge, this is the first attempt to systematically address the problem of identifying signaling modules through integration of the two networks. We now describe the basic procedures in identifying hetero-regulatory modules (HeR module). Briefly, we first define the hetero-regulatory similarity score (HeRS score) that measures the co-function potential of one kinase/phosphatase(KP) and one TF. Then for each pair of TF-(Kinase/Phosphotase), we use their HeRS score as the entry of the HeRS matrix (Figure 3A–B), which represents the similarity between TFs and Kinase/Phosphotase. Through clustering the HeRS matrix, we can identify hetero-regulatory modules in which TFs and Kinases/Phosphotases share similar targets (Figure 3C).

The HeRS score is defined as the Pearson correlation coefficient of the signature profile of a kinase/phosphatase and the binding profile of a TF. Assuming that the transcriptional response to deletion of a kinase/phosphatase is mediated by TFs that function in the same pathway, a high correlation is expected for heterogeneous regulators within pathway. The definition is inspired by the above network motif analysis. In phosphorylation network, where linear regulatory model applies (Figure 1C), the signature profile better characterizes the regulatory role of a kinase/phosphatase than its binding profile. Conversely, transcriptional regulatory network is enriched with parallel regulatory model (Figure 1D). As a result, a regulatory target of a TF is often missing in its signature, but present in the binding profile.

Among the large scale datasets available, we choose KPFN (which provides the signature profile of the kinase/phosphatase) and TFBN (while provides the binding profile of the TF) for integration according to the following reasons: (1) KPFN achieves the best accuracy in predicting co-functional pairs among the phosphorylation datasets, and TFBN is the best among transcriptional regulatory datasets; (2) The transcriptome can serve as an anchor in coupling phosphorylation events with transcriptional regulation, since any change in the transcriptome can be traced back to transcriptional regulation. Among the large scale datasets available, only KPFN characterizes the phosphorylation network at transcriptome level, while KBN and KPIN are at proteome levels. (3) The signature of a TF is usually reduced compared to the actual regulatory targets due to buffering effect. As a result, inference based on TFFN without considering its cellular context can be misleading. Conversely, the binding profile is an unbiased set of potential target genes, and is demonstrated to be a better representative of the transcription factor's function. In addition, a comparison between TFFN and TFBN was conducted, and the HeRS score based on TFBN is proved more accurate in predicting co-functional heterogeneous pairs than that based on TFFN (Figure S3).

Next, we identified hetero-regulatory modules (HeR module) using a clustering approach. A hetero-regulatory module is a set of kinases/phosphatases (KPs) and transcription factors which share targets at the transcriptome level. Mathematically, a cluster of KPs and a cluster of TFs form a HeR module if they have high HeRS scores with each other. Biologically, a HeR module is a set of functionally relevant KPs and TFs, in which KPs transmit signals among each other and regulate the transcriptome through TFs. In another word, a HeR module is a set of regulators, and it is very different from co-regulated gene modules derived from common cluster analysis of microarray datasets, which is a set of co-regulated targets.

Given the hetero-regulatory matrix, in which the element (i, j) represents the hetero-regulating similarity of kinase i and TF j, KPs and TFs are clustered separately by hierarchical clustering. In a HeR module, heterogeneous regulators stand for the corresponding KPs and TFs. The target set of a HeR module is naturally derived, composed of genes that are present in the signature of a KP and bound by a TF in this module (Figure 3A). We applied the procedure to identify HeR modules through integration of KPFN and TFBN in *S.cerevisiae* (see Materials and methods, Figure S4, Table 1), and evaluated the results. To our delight, the HeR modules can be neatly mapped to MAPK pathways. The structure and function of MAPK pathways, as well as its complexity, is well studied in *S.cerevisiae*. Therefore, we take it as a model system to illustrate how HeR modules shed light on the structure and functions of signaling pathways, the cross talk between pathways, and how new functional links are inferred.

**Table 1:** The predicted HeR modules.

HeR Modules	Kinase/Phosphotase	Corr:K/P	TFs	Corr:TF	Ave. Score
1	BCK2/SLT2	0.73	Rlm1	-	0.08
2	BCK2/SLT2	0.73	Nrg1	-	0.07
3	Chk1	-	Adr1/Hsf1	0.79	0.11
4	Cka1	-	Ppr1/Cat8	0.99	0.14
5	Cla4	-	Met28/Met31	0.91	0.09
6	Cmk1/Cmk2/Rim15	0.86	Zap1/Rgm1	0.87	0.09
7	Cmk1/Cmk2/Rim15/Ssk22	0.79	Zap1	-	0.09
8	Dun1/Elm1	0.82	Hot1/Sko1	0.86	0.09
9	Dun1/Elm1	0.82	Cad1	-	0.07
10	Fus3	-	Tec1/Ste12	0.915	0.11
11	Hog1/Pbs2/Ssk2	0.82	Tec1/Ste12	0.915	0.07
12	Hog1/Pbs2/Ssk2	0.82	Sok2/Sko1/Hot1	0.86	0.11
13	Hog1/Pbs2/Ssk2	0.82	Rlm1	-	0.07
14	Ire1	-	Adr1/Hsf1	0.79	0.11
15	Kin3	-	Tec1/Ste12	0.915	0.08
16	Mih1	-	Yhp1/Gsm1	0.75	0.09
17	Sky1	-	Met28/Met31	0.91	0.07
18	Ste20/Ste11/Ste7	0.8	Ste12/Tec1	0.915	0.07
19	Ste20/Ste11/Ste7	0.8	Mcm1/Dig1	0.72	0.07
20	Yck3	-	Arg80/Arg81	0.75	0.08

Kinase/Phosphotase: Kinases or phosphotases in the HeR module.

Corr:K/P: Average correlation coefficient 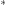 of the kinases/phospotases in one cluster.

TFs: TFs in the HeR module.

Corr:TF: Average correlation coefficient 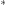 of the TFs in one cluster.

Ave. Score: Average HeRS score of the HeR module.

* : The clustering process is performed using Cluster 3.0.

### Hetero-regulatory modules can recover the structure and function of known signaling pathways

**Figure 4:**
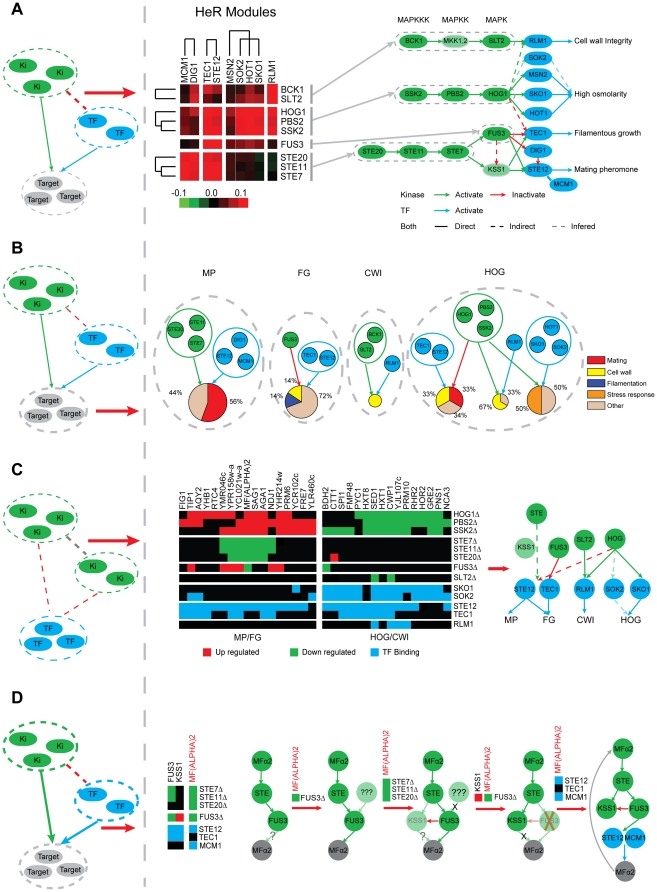
Hetero-regulatory modules inference results. (A) The predicted hetero-regulatory modules recover known MAPK pathways in *S.cerevisiae*, including filamentous growth (FG) pathway, mating pheromone (MP) pathway, cell wall integrity (CWI) pathway, and high osmolarity glycerol (HOG) pathway. (B) Distribution of function of HeR module's target genes. The size of the pie, which represents the functional distribution of the corresponding target gene set, is proportional to the number of target genes in the module. (C) Analysis of the target genes of HOG kinase (Ssk2, Pbs2, Hog1) related HeR modules reveals cross-talk between HOG pathway and other MAPK pathways, and indicates potential role of Sok2 in HOG pathway. (D) HeR modules related to transcription factors Tec1 and Ste12 inferred a feedback loop in mating pathway. Shown is the logic of the inference.

By linking phosphorylation events with transcriptional regulation, HeR modules recover several MAPK pathways known in *S.cerevisiae*, including filamentous growth (FG) pathway, mating pheromone (MP) pathway, cell wall integrity (CWI) pathway, and high osmolarity glycerol (HOG) pathway, mainly due to the high HeRS scores between the KPs and TFs in the MAPK pathways (Figure 4A). For example, Ste7, Ste11, and Ste20 are shared kinase in the upstream of FG and MP pathway, and they form a tight cluster with TFs Ste12, Tec1, Mcm1, and Dig1, which also function in the two pathways. Kinase Fus3 is an inhibitor of the FG pathway, and it forms a HeR cluster with main FG pathway TFs, Tec1 and Ste12. Similarly, kinase in the HOG pathway, Hog1, Pbs2, and Ssk2, are clustered with TFs Hot1 and Sko1. These TFs are involved in osmotic stress response. In another example, our method places TF Rlm1 in the same cluster with kinase Bck1 and Slt2, which are MAPKKK and MAPK in the CWI pathway, respectively. The high HeRS score suggests Rlm1 is a major TF in the CWI pathway. This is consistent with the finding that CWI pathway stimulates expression of cell wall biosynthesis genes via phosphorylation of TF Rlm1 [18]. These examples show the advantage of HeRS score in coupling phosphorylation network with transcriptional regulatory network, and the efficiency of HeR modules in pathway identification.

Besides linking hetero-regulators, a target set is assigned to a HeR module, which provides the capacity to predict the cellular function of the corresponding module. The target set of one HeR module includes all targets regulated by at least one kinase/phosphotase and at least one TF in this module. For the MAPK related HeR modules, we investigated the functional distribution of their target sets. In most cases the abundant function of the target set is consistent with that of the hetero-regulators (Figure 4B). For example, 56% of the target genes with known function in the MP module are annotated with “mating”; all target genes in the CWI module are annotated with “cell wall”. In addition, the kinase in HOG pathway (Hog1, Pbs2, Ssk2) form three HeR modules with different TFs, each representing different functional aspects of the HOG pathway, and the target genes are also enriched with relative functions. For “HOG Kinase - MP TFs” module, 33% of the target genes are annotated with “mating”, for “HOG Kinase - CWI TFs” module, 67% of the target genes are annotated with “cell wall”, for “HOG Kinase - HOG TFs” module, 50% of the target genes are annotated with “stress response”.

### HeR modules reveal the cross-talk between pathways

Many components are shared across different MAPK pathways, but cells maintain the specificity in response to signals. The mechanism to suppress erroneous cross-talk between pathways is not very clear in spite of intensive study on this subject.

In our prediction, the HOG kinase cluster (Hog1, Pbs2, Ssk2) forms hetero-regulatory modules with several TF clusters besides HOG pathway TFs. These TF clusters are involved in FG(Tec1, Ste12), MP(Ste12) and CWI(Rlm1) pathways separately(Figure 4A–C), which suggests the cross talk between the HOG pathway and these other pathways. To further investigate in more detail these HOG related HeR modules, we examined the target set of the HOG kinase (Hog1, Pbs2, Ssk2). When the HOG kinase are deleted, most of the up-regulated genes are annotated in the MP or FG pathway, while the down-regulated genes are mostly annotated in HOG or CWI pathway. The up-regulated genes can only be bound by MP/FG TFs (Ste12, Tec1), while the down-regulated genes are mainly bound by HOG TFs or CWI TFs. These observations suggest that HOG kinase suppresses the cross talk between the HOG pathway and the MP/FG pathway by inhibiting TF activity of Ste12 and Tec1, and induces cross-talk between the HOG and CWI pathways through the activation of Rlm1(Figure 4C).

In fact, there are some experimental evidences supporting our inference. A recent study demonstrated the HOG signaling probably indirectly interrupts signaling transduction in the FG pathway between phosphorylation of Kss1(the MAPK in FG pathway) and activation of Tec1 [19] [20]. Plus, the HOG and MP pathways are likely insulated from each other by specific scaffolds, although whether this is sufficient to prevent inappropriate cross-talk is not clear [20]. Ultimately, it has been found that the HOG pathway could also induce Slt2 through the transcriptional factor Rlm1, which induced the cross talk between the HOG and the CWI pathways [21].

**Figure 5:**
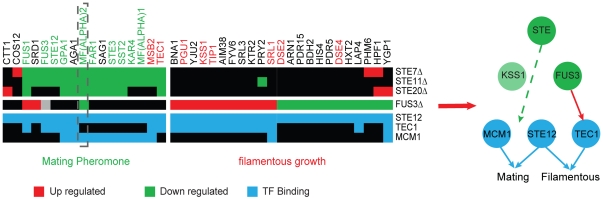
Fus3 inhibits filamentous pathway mainly through inactivating Tec1. Target genes with known function in the two mating pathway kinase related HeR modules, (KP: Ste7, Ste11, Ste20; TF: Ste12, Tec1) and (KP: Fus3;TF: Ste12, Tec1) are shown. The targets of STEs (Ste7, Ste11, Ste20) are enriched with mating pathway genes (green), while the targets of Fus3 are enriched with filamentous pathway genes (red). Deletion of STEs will lead to down-regulation of mating pathway genes, and most of them could be bound by Ste12 and Mcm1 as expected. Deletion of Fus3 mainly up-regulates filamentous pathway genes, which are binding targets of Ste12 and Tec1. Other two filamentous pathway related genes, Dse2 and Dse4, are down-regulated upon Fus3 deletion, and they are inhibitors of filamentous pathway [[32]].

A similar analysis revealed cross-talk between the MP and FG pathways based on two relevant HeR modules((KP: Ste7, Ste11, Ste20; TF:Ste12, Tec1), (KP: Fus3; TF: Ste12, Tec1)). All kinase in these two HeR modules participate in the MP pathway. It is found that the deletion of STEs (Ste7, Ste11, Ste20) mainly induced down-regulation of MP pathway genes, and the deletion of Fus3 caused the up-regulation of FG pathway genes. In addition, TF Ste12 binds to almost all targets of the two HeR modules, but Tec1 mainly binds to targets of Fus3. Mcm1, a MP pathway-specific TF, also forms a HeR module with the STE kinase (KP: Ste7, Ste11, Ste20; TF: Mcm1, Dig1), and it mainly binds to targets of STEs. (Figure 5). These results indicate that MP kinase Fus3 suppresses the cross talk between the MP and FG pathways by suppressing Tec1, and Ste12 is the common TF of both pathways.

The above analysis also coincides with experimental findings. STEs could regulate both MP and FG pathways. However, in non-inducing conditions, the MP pathway is activated, while the FG pathway is suppressed. The specificity is decided by Fus3. Fus3 activates the MP specific genes and inactivates the FG pathway by suppressing Tec1, which is the major TF in the FG pathway. Hence, deletion of STE kinase will only influence genes in the MP pathway but not those in the FG pathway. When Fus3 is knocked out, another kinase, Kss1, will become up-regulated and could partially take over the role of Fus3 to activate Ste12. However, it will not inactivate Tec1 as Fus3 does. As a result, the expression of the MP pathway genes is not sensitive to Fus3 deletion, while FG pathway genes are activated since Tec1 is activated. [18] [20].

### Novel function of Sok2 can be inferred from HeR modules

We have demonstrated that hetero-regulators in the same signaling pathway tend to have a high HeRS score, and HeR modules map well to known pathways. Conversely, a high HeRS score can indicate a co-pathway relationship of the corresponding hetero-regulators. Here, we take TF Sok2 as an example to illustrate how to predict gene functions based on HeR modules. Sok2 forms a HeR module with known HOG TFs and HOG kinase (Figure 4A, Table 1), and it binds to many genes in the HOG pathway (Figure 4B). These data predicts Sok2 as a potential TF in the HOG pathway. Although no previous study has reported Sok2's function in HOG pathway, there is indirect evidence to support this claim.

First, in our analysis, Msn2, Sok2, and HOG TFs (Hot1 and Sko1) form a TF cluster (Figure 4A), and the HeRS scores between Sok2 and HOG kinase are greater than that of the Msn2 and HOG kinase. Since Msn2 is the substrate of Hog1 [22], the above data indicates close relationship between Sok2 and HOG pathway. Plus, in meiosis and mitosis, Sok2 associates with Msn2/4, and they are co-regulated in the cAMP-dependent protein kinase signal transduction pathway [23]. These observations suggest that Sok2 and Msn2/4 may also be co-regulated in HOG pathway. Second, a recent comprehensive phenotypic analysis has found that the deletion of Sok2 caused a decrease in the hyperosmotic stress resistance of cells [24]. This provides direct evidence that Sok2 is involved in HOG pathway.

Both our results and experimental data suggest Sok2 has an extensive interaction with HOG pathway, and may be a novel transcription factor in this pathway.

### A potential feedback loop in mating pathway is predicted

Fus3 and Kss1 are paralogs, and they are redundant MAPKs in the MP pathway. Previous studies reported that the redundancy of Fus3 and Kss1 is partial, since deletion of Fus3 resulted in a 10% reduction in mating efficiency compared to the wild type level, but the deletion of Kss1 has virtually no effect [25] [26]. The phenomenon of partial redundancy is lacking in explanation, while an in-depth examination of the HeR module reveals the missing link.

Two HeR modules are related to the MP pathway, HeR module FUS3 (KP: Fus3; TF: Tec1, Ste12) and HeR module STE (KP: Ste20, Ste11, Ste7; TF: Tec1, Ste12). As analyzed above, genes in MP pathway should be down-regulated upon deletion of the STE kinase, but not sensitive to the deletion of Fus3. When we compared the target sets of Fus3 and STE, all genes behave as expected except for MF(ALPHA)2, which is down-regulated upon deletion of both Fus3 and STE kinase (Figure 5). Interestingly, MF(ALPHA) 2 is the upstream signal (alpha factor) of the mating pathway, which activates the STE kinase. A simple analysis based on these observations could illustrate a positive feedback loop including Fus3 but not Kss1 (Figure 4D). In addition, MF(ALPHA)2 is occupied by Mcm1 and Ste12 but not Tec1, which is further evidence that MF(ALPHA)2 is transcriptional regulated in the mating pathway. The deletion of Fus3 leads to a decreased expression level of MF(ALPHA)2, resulting in the positive feedback being cut off. In contrast, the deletion of Kss1 does not affect the activity of MF(ALPHA)2, allowing the positive feedback loop to be retained. Since another mating pheromone alpha factor MF(ALPHA)1 is more highly expressed and produces most alpha-factor, deprivation of this positive feedback explains the slight reduction (10%) of mating efficiency.

## Discussion

Phosphorylation and transcriptional regulatory networks work in coordination in response to stress and changing environments. Experimental and computational methodologies needed to dissect each of these two networks are largely available in model organism *S.cerevisiae*. However, it is still difficult to determine the transcription factors that respond to a specific kinase/phosphatase. Our results show that the functional link between these two kinds of regulators can be accurately predicted by a hetero-regulatory similarity score, computed from the comparison of their regulatory profiles. We tried several statistics to measure the similarity between regulatory profiles, including Pearson correlation coefficient, topological overlap matrix [27], Jaccard index [28], and cumulative hyper-geometric density [29]. Pearson correlation coefficient was chosen since it performed better than the other statistics. In particular, several hetero-regulatory modules are predicted from the clustering analysis, and these modules recover known MAPK pathways in *S.cerevisiae*. Another major contribution of this study is that we demonstrate phosphorylation and transcriptional regulatory networks differ in motif usage, and the predictive power of the functional signature and physical interaction profile of a regulator is dependent on its local topology. In particular, the “regulator chain” motif is abundant in phosphorylation networks, suggesting these networks are largely characterized by linear signal transduction. Hence, kinase/phosphatase regulators in a regulator chain share a similar functional signature while possessing a divergent binding profile. However, the story is different for transcriptional regulatory networks. Due to the frequent use of “bi-component” motifs and the backup effect between transcription factors, the functional signature of transcription factors is hardly predictive. Instead, the binding profile of a transcription factor better represents its function. In light of these observations, we defined the hetero-regulatory similarity score, which couples phosphorylation networks with transcriptional regulatory networks. In utilizing this scoring methodology, hetero-regulatory modules that link kinase/phosphatase and transcription factors can be identified through the computational integration of individual networks without requiring labor-intensive screening experiments. It is also worth noticing that network topology is an important factor when choosing an appropriate method to analyze the network.

One limitation of our method is that the functional link of some kinase/phosphatase cannot be predicted because they have no signature. In other words, the expression level of downstream genes do not change upon deletion of the corresponding regulator. One explanation of this phenomenon is the buffering effect. For example, Mkk1 and Mkk2 are two redundant MAPKKs involved in the protein kinase signaling pathway that controls cell integrity. The deletion of either kinase has no significant effect, since the other one will take over its entire function. Another reason is that some kinase are inactive in normal conditions. This limitation can be compensated by measuring the expression level under conditions of induced stress or by constructing double mutation strains in which functionally redundant kinase are deleted simultaneously. Our method will gain additional power when such data becomes available.

Deciphering the signal transduction in normal cells and cancer cells is an essential step in curing cancer. In spite of its importance, understanding of the human signaling pathways is very limited. In fact, predicting the regulatory relationship between kinases/phosphatases and transcription factors remains an extremely difficult problem. The methodology proposed in this study with hetero-regulatory similarity score and the hetero-regulatory module attempts to solve this problem. Our results demonstrate that signal transduction can be accurately recapitulated by a multi-level analysis of large-scale datasets. Although this study is conducted and tested in the model organism *S.cerevisiae*, we suppose that this method can be easily exploited in other organisms when the data becomes available. Currently, the binding specificity of many transcription factors has been studied through ChIP-chip and ChIP-seq experiments in human. With the recent development in RNAi technology, the construction of kinase/phosphatase single mutation cell lines and genome-wide measurement of gene expression level in these cell lines will be straightforward. Thus, our approach serves as a promising tool for the discovery of signaling pathways in human.

## Materials and Methods

### Materials

Five networks were used in this study: KPFN, a functional network derived from a microarray study of kinase/phosphatase single deletion strains [4]; TFFN, a functional network derived from TF single deletion strains [5]; KBN, a biochemical network derived from *in vitro* protein chip [1]; KPIN, physical interaction network of kinase/phosphatase interaction [10]; TFBN, a TF-DNA interaction network derived from ChIP-chip experiments [12] [13]. The datasets of KPFN, TFFN, KBN and KPIN were downloaded from the supplementary of the original papers, and we adopted the threshold for regulatory relationship as used by the original authors. TFBN was downloaded from YEASTRACT: http://www.yeastract.com/. Each network was represented by a binary matrix (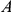), where the rows and columns correspond to target genes and regulators respectively. 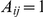 if regulator j regulates target i; 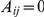, otherwise. The gold standard co-functional gene pairs are manually curated by biological experts [30], which can be downloaded from the supplementary of the original paper.

### Prediction of co-functional gene pairs

For each network, the functional relevance of two genes is calculated using the Pearson correlation coefficient (PCC) between interaction profiles of two regulators (columns). Gene pairs are ranked descending by PCC, and a percent of top-ranking pairs are predicted as co-functional. The true positive rate at various percent levels is calculated and compared across the five networks.

### Motif enrichment analysis

In order to evaluate the enrichment of network motifs, their occurrence is calculated and compared with randomly shuffled networks similar to a previous study [31]. During the randomization steps, the degree of genes is preserved. In detail, to generate such a random network, we performed “permutations” of the real networks (Figure S5). For KBN and TFBN, 1000 randomized networks were generated, and the edges of each network were shuffled 5000 times. We assigned a p-value to a network motif according to the fraction of randomized networks in which the motif occurs more frequently than the real network. The Z-score of a motif is calculated as the difference of its observed occurrence in the real network and its averaged occurrence in the 1000 random networks, normalized by the estimated standard deviation. In order to randomly shuffle the combined network, KBN and TFBN were first shuffled separately 5000 times, and then combined to form a randomized combined network. P-values and Z-scores are similarly derived from 1000 randomized combined networks.

### Identification of Hetero-regulatory modules

Given the hetero-regulatory score matrix(R pseudo-code see Figure S6), the goal is to detect sub-matrixes that satisfy the following criterias: (1) TFs in a module are similar in their HeRS score profiles; (2) KPs in a module are similar in their HeRS score profiles; (3) TF and KP have high HeR score with each other. A heuristic strategy was employed to identify hetero-regulatory modules in the integrated network. We started by hierarchically clustering the KPs and TFs respectively(Figure S4). The dendrogram is cut arbitrarily at PCC level 0.7, thus deriving several KP clusters and TF clusters. In order to allow multiple membership of a regulator, HeR modules are detected from two directions. First, for a KP cluster, a TF that has a HeRS score greater than a certain threshold T with all KPs in the cluster is added to form a candidate HeR module. Two candidate HeR modules are merged if they share a KP cluster and the PCCs of their TFs are greater than 0.7. Similarly, HeR modules can be built by adding KPs to TF clusters with the above rules. Thus, the resulting modules contain at least two KPs or two TFs, and a KP or TF can be involved in several HeR modules. The threshold T is set to 0.055, resulting in 23 HeR modules (Table 1). The threshold is chosen by supervision that allowed the known MAPK pathways to be identified. However, the result is not sensitive to the threshold since the HeR scores between hetero-regulators in the same MAPK pathway rank top in the HeR matrix, and KPs in a kinase cascade form a tight cluster with each other.

## Supporting Information

Figure S1
**One example of bi-component loops motif in transcriptional regulatory network, Ste12 and Tec1.** Ste12 and Tec1 both regulate Kss1 which is a kinase in the filamentous growth pathway. Ste12 activate the mating pathway gene Ste3, while Tec1 does not. G1 and G2 are other genes in the yeast genome which are not regulated by Ste12 and Tec1. Theoretically and experimentally, single deletion of Ste12 will decrease the expression level of Ste3, and the deletion of Tec1 has no observable effect because of the genetic buffering with Ste12 [[33]]. In this case, similarity in physical binding profile (cor
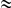
0.58) can reveal the close relationship between Ste12 and Tec1, while functional interaction profiles (cor
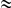
0) cannot.(TIF)

Figure S2 **Global topological properties of KBN and TFBN.** In both networks, the degree distributions obey a power law form.
(TIF)

Figure S3 **Comparison of prediction accuracy of hetero-regulatory scores derived from TFFN and TFBN.** The HeRS score based on TFBN is proved more accurate in predicting co-functional heterogeneous pairs than that based on TFFN.
(TIF)

Figure S4 **Hierarchical clustering of the hetero-regulatory similarity matrix.** The clustering was performed on HeRS matrix using Cluster 3.0. The options used were “Complete Linkage”, and processed simultaneous for the columns(TFs) and rows (Kinases/Phosphotases). The cutoff of significant Pearson correlation coefficient(PCC) is set to 0.1.
(TIF)

Figure S5 **Permutation procedure in generating the random network.** As shown, regulatory gene pairs like (R1,T1) and (R2,T2) are randomly chosen, then the edges are permutated.
(TIF)

Figure S6 **The pseudo-code for R.**
(TIF)
