# Supplementary figures and images for: Integrating Phosphorylation Network with Transcriptional Network Reveals Novel Functional Relationships
Source: PLoS One. 2012 Mar 14;7(3):e33160. doi: 10.1371/journal.pone.0033160 (PMC3303811; doi:10.1371/journal.pone.0033160)

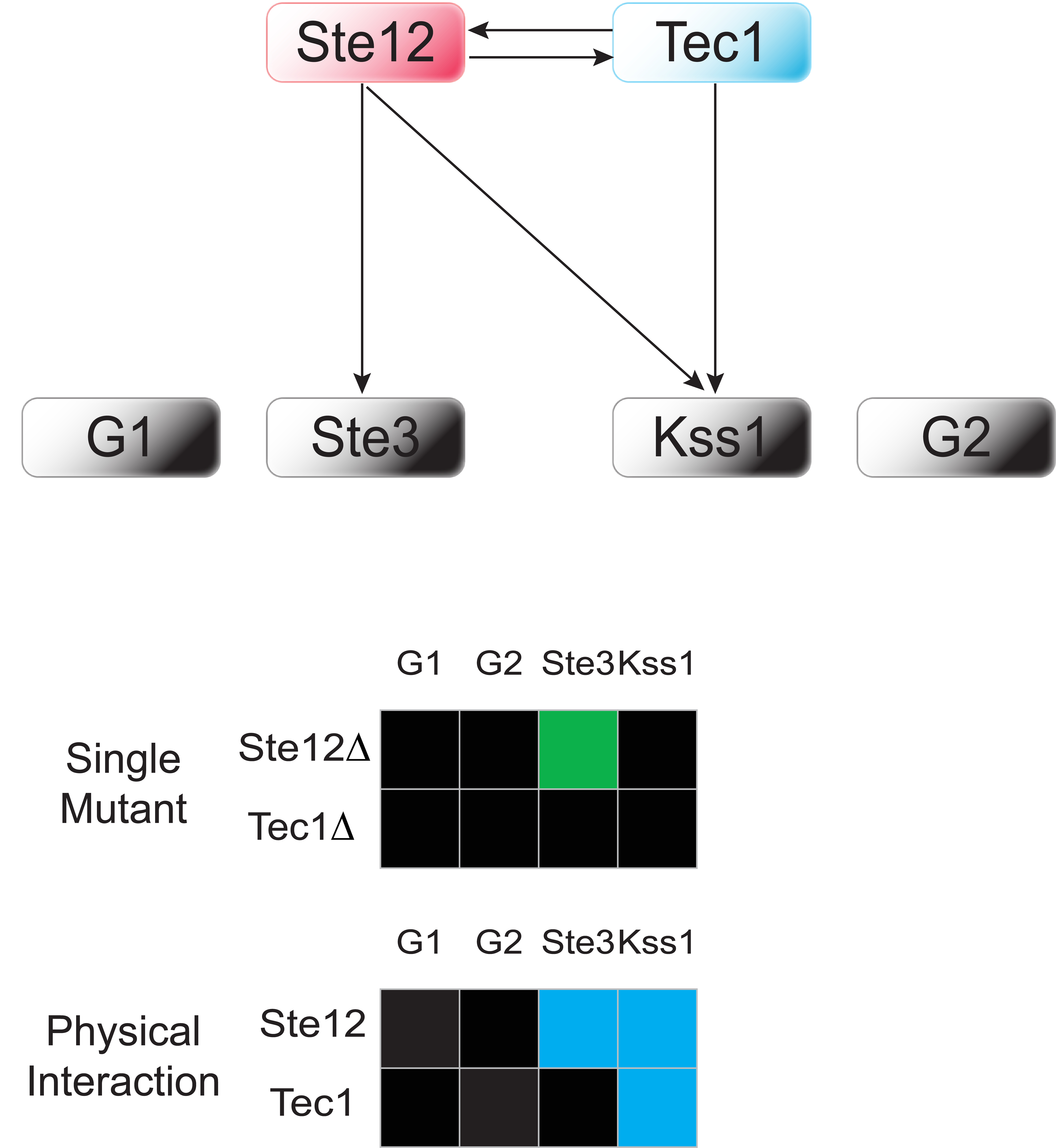

Supplement: Figure S1 — One example of bi-component loops motif in transcriptional regulatory network, Ste12 and Tec1. Ste12 and Tec1 both regulate Kss1 which is a kinase in the filamentous growth pathway. Ste12 activate the mating pathway gene Ste3, while Tec1 does not. G1 and G2 are other genes in the yeast genome which are not regulated by Ste12 and Tec1. Theoretically and experimentally, single deletion of Ste12 will decrease the expression level of Ste3, and the deletion of Tec1 has no observable effect because of the genetic buffering with Ste12 [[33]]. In this case, similarity in physical binding profile (cor0.58) can reveal the close relationship between Ste12 and Tec1, while functional interaction profiles (cor0) cannot. (TIF) [file pone.0033160.s001.tif]

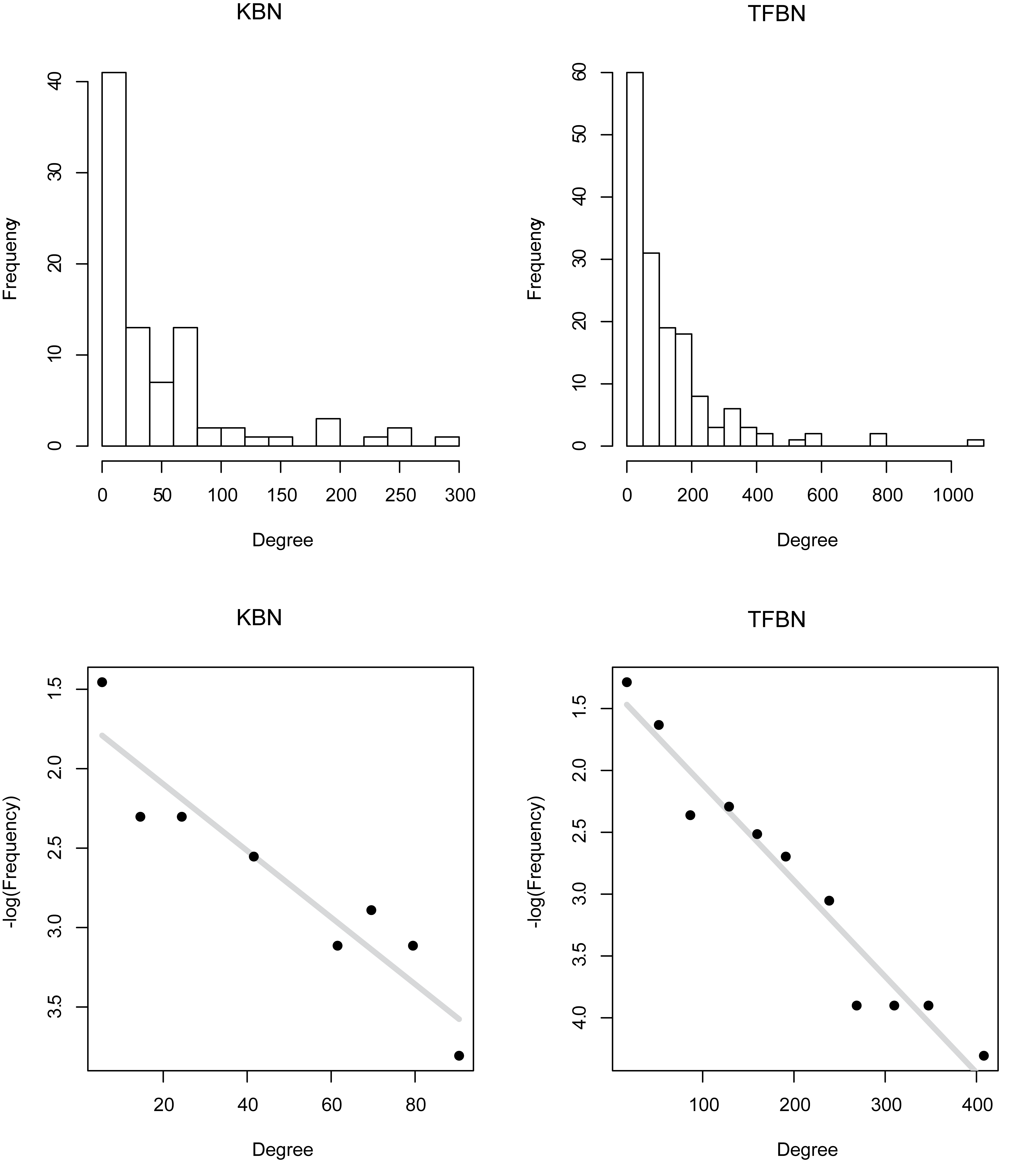

Supplement: Figure S2 — Global topological properties of KBN and TFBN. In both networks, the degree distributions obey a power law form. (TIF) [file pone.0033160.s002.tif]

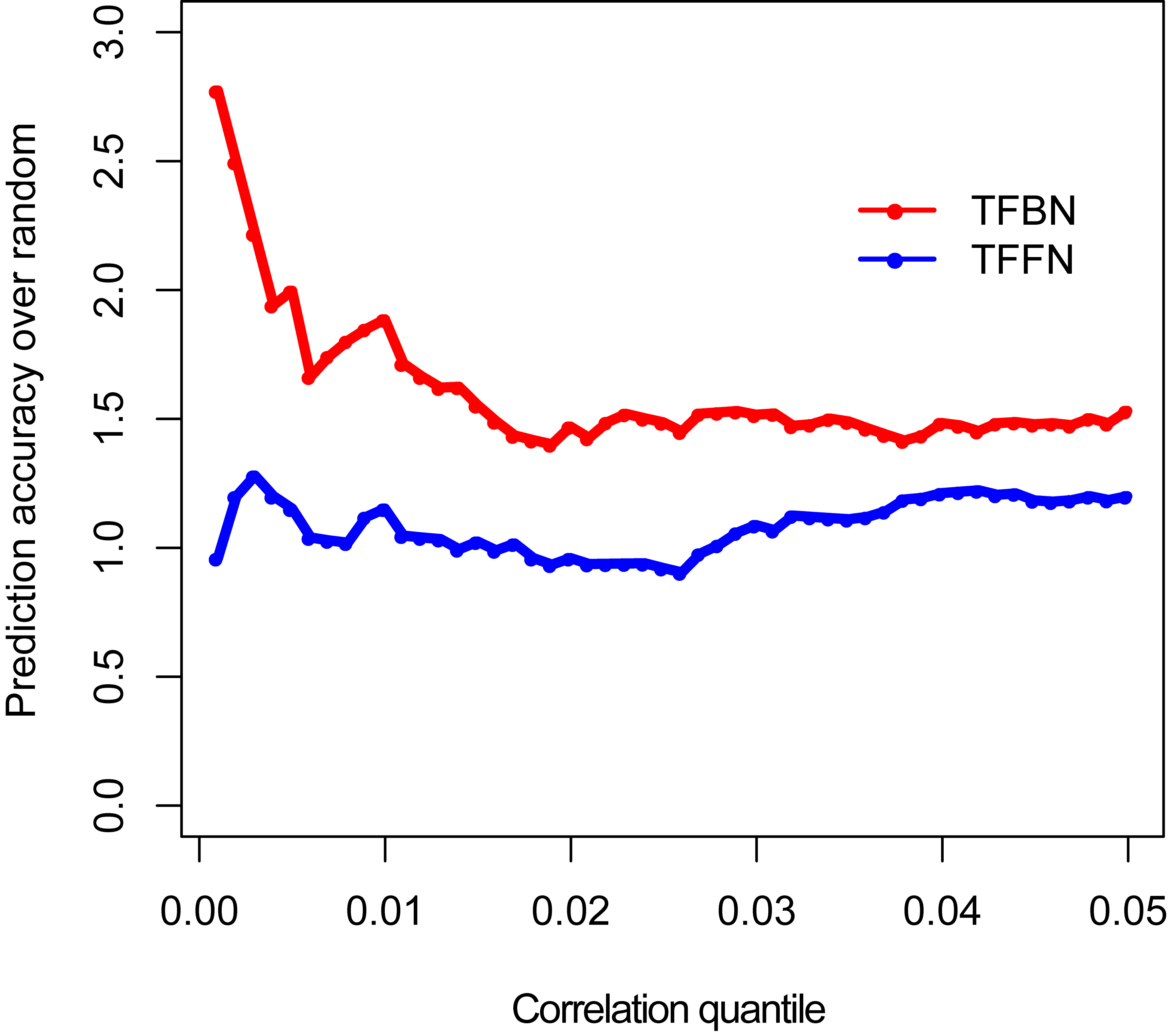

Supplement: Figure S3 — Comparison of prediction accuracy of hetero-regulatory scores derived from TFFN and TFBN. The HeRS score based on TFBN is proved more accurate in predicting co-functional heterogeneous pairs than that based on TFFN. (TIF) [file pone.0033160.s003.tif]

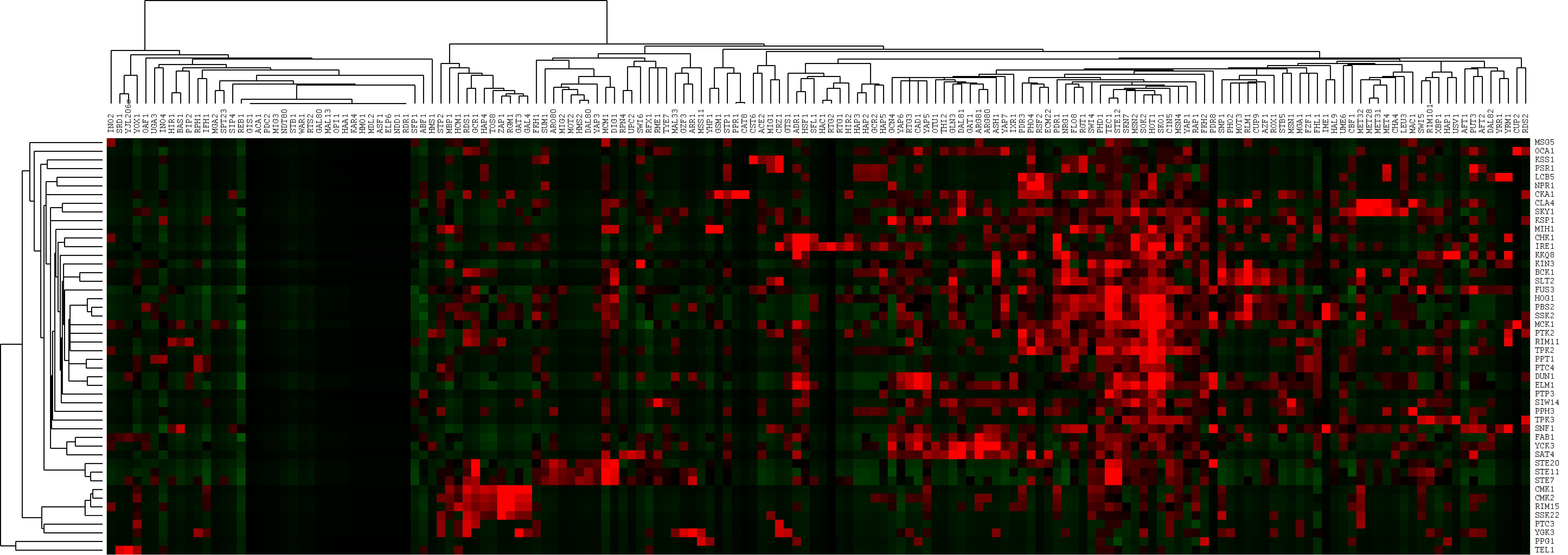

Supplement: Figure S4 — Hierarchical clustering of the hetero-regulatory similarity matrix. The clustering was performed on HeRS matrix using Cluster 3.0. The options used were “Complete Linkage”, and processed simultaneous for the columns(TFs) and rows (Kinases/Phosphotases). The cutoff of significant Pearson correlation coefficient(PCC) is set to 0.1. (TIF) [file pone.0033160.s004.tif]

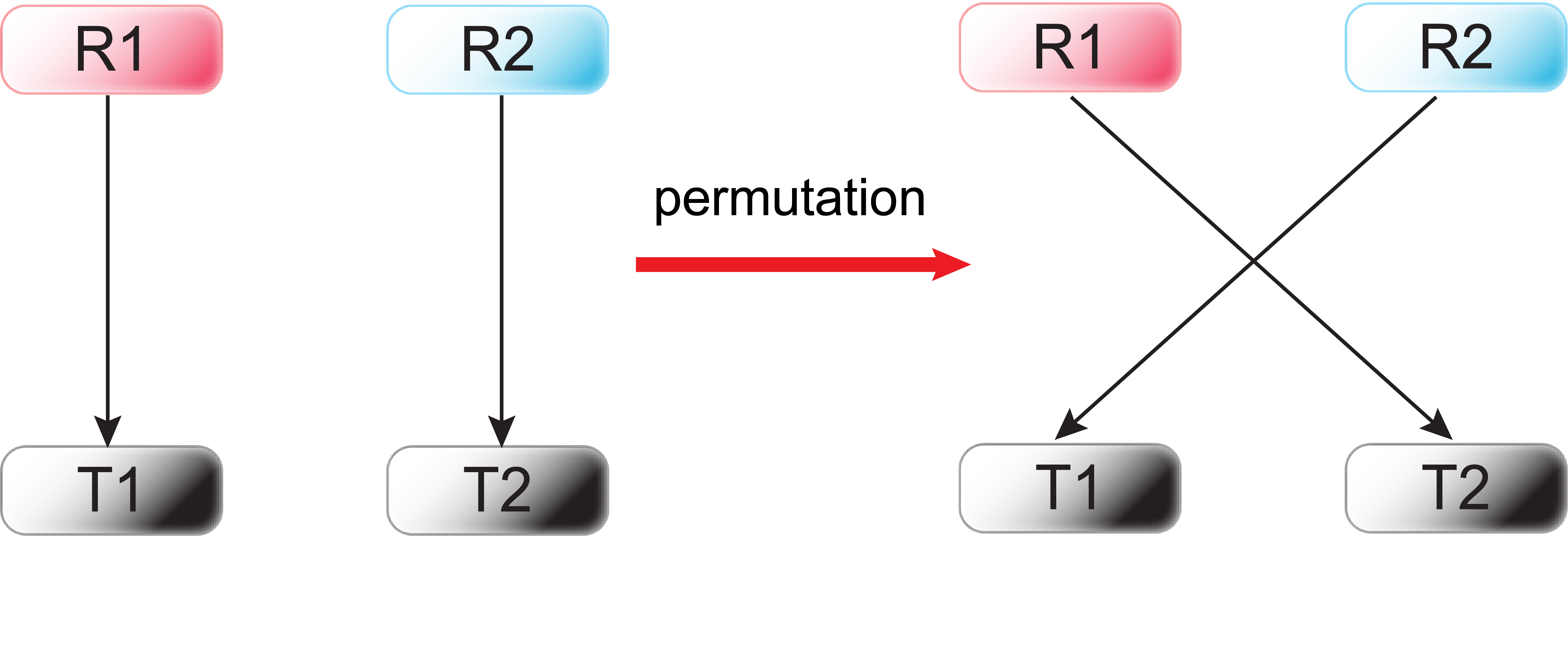

Supplement: Figure S5 — Permutation procedure in generating the random network. As shown, regulatory gene pairs like (R1,T1) and (R2,T2) are randomly chosen, then the edges are permutated. (TIF) [file pone.0033160.s005.tif]

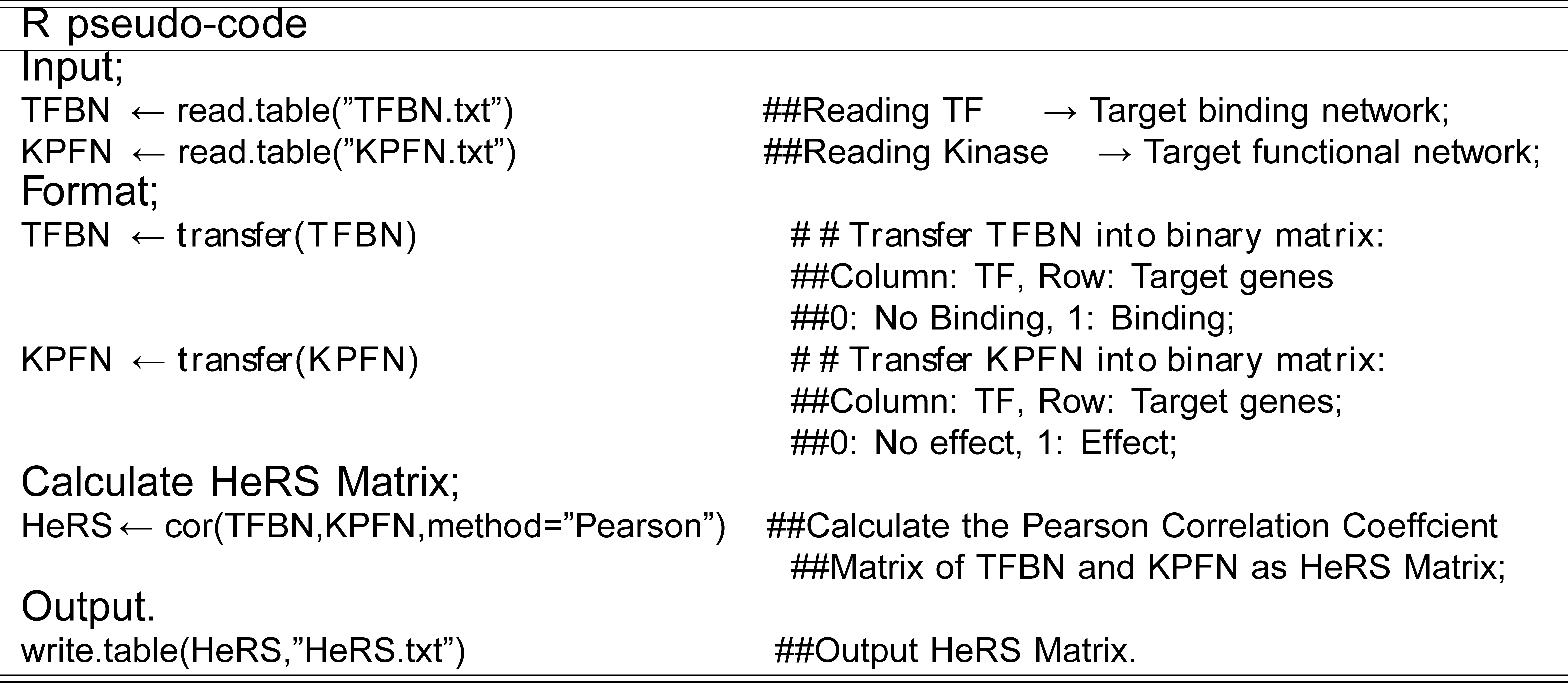

Supplement: Figure S6 — The pseudo-code for R. (TIF) [file pone.0033160.s006.tif]
